# Hepatitis C in men who have sex with men in London – a community survey

**DOI:** 10.1111/hiv.12050

**Published:** 2013-06-18

**Authors:** H Price, R Gilson, D Mercey, A Copas, J Parry, A Nardone, A Johnson, G Hart

**Affiliations:** 1 Research Department of Infection and Population Health, University College LondonLondon, UK; 2 Health Protection AgencyLondon, UK

**Keywords:** community survey, hepatitis C, HIV, prevalence

## Abstract

**Objectives:**

For the last 10 years there has been an epidemic of hepatitis C virus (HCV) infection in men who have sex with men (MSM) in Europe, North America and Australia. The majority of those infected are also HIV-positive and it is unclear to what extent HIV-negative MSM are also at increased risk of infection with HCV. This study provides the first examination of the association between HIV and hepatitis C serostatus in a sample of MSM recruited in community settings.

**Methods:**

A total of 1121 participants completed a short questionnaire in 2008/2009 giving demographic and behavioural data, and donated a sample of oral fluid that was subsequently tested for antibodies to selected pathogens (HIV, syphilis and HCV).

**Results:**

The seroprevalence of hepatitis C antibody was 2.1% [95% confidence interval (CI) 1.4–3.2%]. It was more common in those with HIV infection [7.7% (95% CI 4.2–12.9%) *vs.* 1.2% (95% CI 0.6–2.1%) in those without HIV infection; *P* < 0.001], those with a history of syphilis [12.2% (95% CI 4.6–24.8%) *vs.* 1.7% (95% CI 1.0–2.6%) in those without such a history; *P* < 0.001] and those who reported casual unprotected anal intercourse in the previous year [4.1% (95% CI 2.0–7.4%) *vs.* 1.2% (95% CI 0.5–2.2%) in those who did not report such intercourse; *P* = 0.01]. There was no relationship between hepatitis C antibody (anti-HCV) status and other demographic variables (age, ethnicity, employment status or education).

**Conclusions:**

The seroprevalence of anti-HCV in HIV-negative MSM (1.2%) was higher, but not significantly higher, than that in the general population (0.67%). The prevalence was significantly higher in those infected with HIV or with previous syphilis infection and in those reporting unprotected anal intercourse. Our findings support current British Association for Sexual Health and HIV guidelines recommending the provision of selective HCV testing in MSM according to individual risk profile.

## Introduction

In the UK, most cases of hepatitis C are contracted through injecting drug use [1] but since around 2000 there has also been an epidemic in HIV-positive men who have sex with men (MSM) [2]. This epidemic initially appeared to be restricted to Europe [3–5] but has since spread to North America and Australia.

There is conflicting evidence of the degree to which there has been spread to HIV-negative MSM [6–8]. Of the first 105 cases reported by genitourinary medicine (GUM) clinics between January 2008 and May 2009 to The Enhanced Surveillance of Newly Acquired Hepatitis C Infection in MSM (SNAHC), 96% were already diagnosed as HIV positive [9].

The latest estimate (for 2005) of the prevalence of anti-hepatitis C virus (HCV) antibody (anti-HCV) in the general population of England aged 15 to 59 years is 0.67% [10]. Studies in central London GUM clinics have found the prevalence of anti-HCV among HIV-negative MSM to be 0.61–0.7% [7,8], while in HIV-positive MSM the prevalence nationally is estimated to be 7.2% [11]. To our knowledge, there has been no prior estimate of the community prevalence of HCV antibody in MSM.

Hepatitis C testing is not universal for HIV-negative MSM attending GUM clinics. The British Association for Sexual Health and HIV UK National Guideline on the Management of the Viral Hepatitides A, B & C 2008 [12] states that testing can be considered in MSM. This study evaluates the current prevalence of hepatitis C antibody in a community sample to determine whether this guideline should be revised.

## Methods

The Gay Men's Sexual Health Survey was first carried out in 1996 [13]. Its purpose is to investigate changes in the sexual risk behaviour for HIV infection of MSM over time. Participants are recruited from gay bars, clubs and saunas across London as part of a repeat cross-sectional survey. The survey consists of an anonymous self-completion paper questionnaire including demographic and behavioural questions. In 2008, an oral fluid sample was collected and tested for antibodies to HIV, HCV and syphilis and the results linked to the anonymous questionnaire.

Testing for the three markers was performed at the Health Protection Agency (HPA), Colindale, London, using the HIV 1+2 IgG antibody capture ELISA (GACELISA), Ortho HCV enzyme-linked immunosorbent assay (ELISA) (Ortho-Clinical Diagnostics Inc., Raritan, New Jersey, USA) and Murex Syphilis immune-capture EIA tests (Diasorin, Saluggia, Italy) [14–16]. HIV 1+2 GACELISA is an in-house test; the HCV and syphilis tests are commercial tests employing procedures developed, optimized and characterized at the HPA for oral fluid testing.

Associations with anti-HCV status were assessed using Fisher's exact test for categorical factors and the Mann–Whitney test for ordinal factors.

Ethical approval for all aspects of the study was obtained from UCL Ethics Committee.

## Results

Questionnaires were received from 1279 MSM, 1121 of whom also gave a sample of oral fluid using the OraSure™ (OraSure Technologies, Inc., Bethlehem, USA) collection device. The median age was 33 years (range 16–81 years). Most (83.8%) were of White ethnicity, 3.4% were Black, 1.9% were South-East Asian, 2.5% were Asian and 8.5% were of other ethnicities.

The prevalence of antibodies to HCV was 2.1% [24 of 1121; 95% confidence interval (CI) 1.4–3.2%].

Anti-HCV was more common in the 168 subjects (15.0%) who tested positive for HIV [7.7% (95% CI 4.2–12.9%) *vs.* 1.2% (95% CI 0.6–2.1%) in those who did not test positive; *P* < 0.001] and in the 49 subjects (4.4%) who tested positive for syphilis [12.2% (95% CI 4.6–24.8%) *vs.* 1.7% (95% CI 1.0–2.6%) in those who did not test positive; *P* < 0.001].

There was no statistically significant association between anti-HCV and age (*P* = 0.24), ethnicity (*P* = 0.66), employment status (*P* = 0.76) or duration of education after age 16 years (*P* = 0.71).

Hepatitis C antibody was more common in those who had attended a GUM clinic in the previous year (3.4% *vs.* 0.9% in those who had not attended a GUM clinic; *P* < 0.01) and in those who reported having a sexually transmitted infection (STI) in the previous year (4.7% *vs.* 1.8% in those who did not report an STI; *P* = 0.03) but not in those who had been tested for HIV in the previous year (1.9% *vs.* 2.2% in those who had not been tested for HIV; *P* = 0.83).

Hepatitis C seropositivity was not associated with reported unprotected anal intercourse (UAI) in the last year (2.9% *vs.* 1.4% for those who did not report UAI; *P* = 0.10) but was associated with UAI with casual partners [4.1% (95% CI 2.0–7.4%) *vs.* 1.2% (95% CI 0.5–2.2%) for those who did not report UAI with casual partners; *P* = 0.01]. It was not associated with reported UAI with a partner of a discordant or unknown HIV status in all participants (4.3% *vs.* 2.1% for no such reported UAI; *P* = 0.26) or in HIV-negative MSM (2.0% *vs.* 1.5%, respectively; *P* = 0.70).

HIV-negative MSM who attended a GUM clinic in the previous year were no more likely to be anti-HCV positive than those who did not attend (1.4% *vs.* 1.0%, respectively; *P* = 0.76).

## Discussion

We found the prevalence of anti-HCV in MSM overall to be 2.1%, which is significantly higher than the 0.67% estimate for those of similar age in the general UK population. Anti-HCV was nearly seven times more common in HIV-positive than in HIV-negative MSM and the prevalence in HIV-negative MSM (1.2%; 95% CI 0.58–2.1%) was not significantly higher than in the general population.

Anti-HCV was also more common in those who tested positive for syphilis or reported an STI in the previous year. It was more common in those reporting UAI with casual partners.

The Gay Men's Sexual Health Survey (2008) did not ask participants about injecting drug use and so we were unable to control for this major risk factor for hepatitis C.

In a community sample of MSM in London we did not find any evidence of a large number of HIV-negative MSM with evidence of exposure to hepatitis C. This supports the practice at most GUM clinics where HCV testing (which should be performed annually in those who are HIV positive and more frequently in those at increased risk of HCV infection) is not routine for HIV-negative MSM. It also suggests that the British Association for Sexual Health and HIV guidelines should not change, and should continue to recommend that HCV testing be considered for MSM, according to risk profile. Those with a history of UAI with casual partners and those with syphilis infection should be considered at higher risk.
